# Comparable blood velocity changes in middle and posterior cerebral arteries during and following acute high‐intensity exercise in young fit women

**DOI:** 10.14814/phy2.14430

**Published:** 2020-04-27

**Authors:** Lawrence Labrecque, Audrey Drapeau, Kevan Rahimaly, Sarah Imhoff, François Billaut, Patrice Brassard

**Affiliations:** ^1^ Department of Kinesiology Faculty of Medicine Université Laval Québec Québec Canada; ^2^ Research Center of the Institut Universitaire de Cardiologie et de Pneumologie de Québec‐Université Laval Québec Québec Canada

**Keywords:** exercise, middle cerebral artery, posterior cerebral artery, women

## Abstract

The cerebral blood flow response to high‐intensity interval training (HIIT) remains unclear. HIIT induces surges in mean arterial pressure (MAP), which could be transmitted to the brain, especially early after exercise onset. The aim of this study was to describe regional cerebral blood velocity changes during and following 30 s of high‐intensity exercise. Ten women (age: 27 ± 6 years; VO_2max_: 48.6 ± 3.8 ml·kg·min^−1^) cycled for 30 s at the workload reached at $\dot{V}$O_2max_ followed by 3min of passive recovery. Middle (MCAv_mean_) and posterior cerebral artery mean blood velocities (PCAv_mean_; transcranial Doppler ultrasound), MAP (finger photoplethysmography), and end‐tidal carbon dioxide partial pressure (P_ET_CO_2_; gaz analyzer) were measured. MCAv_mean_ (+19 ± 10%) and PCAv_mean_ (+21 ± 14%) increased early after exercise onset, returning toward baseline values afterward. MAP increased throughout exercise (*p* < .0001). P_ET_CO_2_ initially decreased by 3 ± 2 mmHg (*p* < .0001) before returning to baseline values at end‐exercise. During recovery, MCAv_mean_ (+43 ± 15%), PCAv_mean_ (+42 ± 15%), and P_ET_CO_2_ (+11 ± 3 mmHg; *p* < .0001) increased. In young fit women, cerebral blood velocity quickly increases at the onset of a 30‐s exercise performed at maximal workload, before returning to baseline values through the end of the exercise. During recovery, cerebral blood velocity augments in both arteries, along with P_ET_CO_2_.

## Graphical abstract

The novel findings of this study are that in young fit women, the acute cerebral blood velocity response to a high‐intensity exercise bout is biphasic and characterized by a rapid increase followed by a return toward baseline values. The subsequent recovery period is also characterized by large cerebral blood velocity elevations.

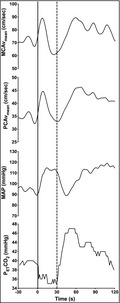

## 1 INTRODUCTION

High‐intensity interval training (HIIT) is a popular method of training, not only to optimize performance in athletes but also within a context of rehabilitation in various clinical populations. HIIT is preferred for its efficacy, cost‐effectiveness, and time saving (Batacan, Duncan, Dalbo, Tucker, & Fenning, 2017). HIIT procures similar or even superior beneficial metabolic and cardiovascular adaptations in untrained individuals, athletes, and clinical populations in comparison with the more traditional moderate‐intensity continuous exercise training (MICT) (Gibala, Little, MacDonald, & Hawley, 2012). Furthermore, cardiovascular risk does not seem to be superior during HIIT compared to MICT in patients with coronary artery disease, heart failure, systemic hypertension, obesity, or with the metabolic syndrome (Weston, Wisloff, & Coombes, 2014; Wewege, Ahn, Yu, Liou, & Keech, 2018). However, whether HIIT poses a risk for the brain remains to be clearly determined. Indeed, repetitive, rapid, and important elevations in blood pressure (BP) induced by short bouts of high‐intensity exercise could be transmitted to the brain vasculature, which in turn may elevate the risk of cerebral hyperperfusion injury (Bailey et al., 2011; Phillips et al., 2018).

HIIT is characterized by the repetition of intense exercise bouts of various duration and length, interspersed with passive or active resting periods (Gibala et al., 2012). The temporal response of cerebral blood flow to a rapid surge in blood pressure (BP) induced by either one high‐intensity exercise bout or a succession of high‐intensity exercise intervals is not well described. To the best of the authors’ knowledge and among all studies interested in the regulation of cerebral blood flow during HIIT, only one study examined cerebral blood velocity (CBV) changes during a single high‐intensity exercise bout (Curtelin et al., 2018). Specifically, during a 30‐s all‐out sprint exercise performed by young healthy participants, Curtelin et al. (2018) reported a 16% increase in middle cerebral artery mean blood velocity (MCAv_mean_) 7.5 s after the onset of the sprint exercise. This MCAv_mean_ augmentation was concomitant to a 16‐mmHg increase in mean arterial pressure (MAP) over the 30‐s sprint, with maximal MAP being reached at the end of exercise. Following this acute high‐intensity exercise bout, MCAv_mean_ showed a biphasic pattern, which began with an abrupt 18% reduction, 2.5 s following the end of exercise. Thereafter, MCAv_mean_ progressively increased to reach maximal values 40 s into recovery before normalization to baseline value at 60 s post‐exercise. Accordingly, the immediate recovery period following an exercise bout performed at high‐intensity represents another period during which the cerebral vessels may be acutely challenged in the presence of transient and important changes in BP.

Of note, only young healthy men were examined by Curtelin et al. (2018). Considering the impact of sex on various cerebral blood flow determinants (Favre & Serrador, 2019; Gur et al., 1982; Kastrup, Thomas, Hartmann, & Schabet, 1997; Labrecque et al., 2019), these findings cannot be directly translated to young healthy women. In fact, although there is a lack of adequately designed studies in the literature, sex could have an influence on CO_2_ reactivity (Kastrup, Happe, Hartmann, & Schabet, 1999; Kastrup et al., 1997; Matteis, Troisi, Monaldo, Caltagirone, & Silvestrini, 1998; Peltonen et al., 2015) and cerebral autoregulation (Deegan, Cooke, Lyons, Olaighin, & Serrador, 2010; Deegan et al., 2011; Favre & Serrador, 2019; Labrecque et al., 2019). The description of the CBV response to a high‐intensity exercise and the following recovery in women is therefore essential. In addition, regional differences exist in the distribution of cerebral blood flow during incremental aerobic exercise to exhaustion (Sato, Ogoh, Hirasawa, Oue, & Sadamoto, 2011; Smith et al., 2012). Specifically, previous findings demonstrated a continuous blood flow elevation in the posterior cerebral circulation, compared to a biphasic blood flow response in the anterior cerebral circulation [reviewed in Smith & Ainslie (2017)]. However, CBV changes during and after high‐intensity exercise has only been examined in the anterior cerebral circulation via insonation of the MCA and no studies have attempted to examine the impact of high‐intensity exercise on the posterior cerebral circulation. This is of importance, since regional differences in key cerebral blood flow determinants (i.e., cerebral autoregulation, cerebrovascular reactivity to CO_2_) could make posterior cerebral regions more susceptible to hyperperfusion injuries during rapid and important surges in BP induced by high‐intensity exercise [reviewed in Lucas, Cotter, Brassard, & Bailey (2015)]. Since our knowledge on cerebrovascular responses induced by high‐intensity exercise is limited, it remains essential to describe cerebrovascular hemodynamic responses to such exercise first, in order to provide a basis for designing more complex cerebrovascular studies involving HIIT.

Therefore, the aim of this study was to describe regional CBV changes during and following an acute bout of high‐intensity exercise in young healthy women. We hypothesized MCAv_mean_ and PCA mean blood velocity (PCAv_mean_) would increase proportionally to MAP in the beginning of the exercise, but would return toward baseline at the end of the exercise bout; the amplitude of change in blood velocity would be of greater amplitude in the PCA than in the MCA and the recovery period following high‐intensity exercise would be characterized by large increases in MCAv_mean_ and PCAv_mean_.

## 2 MATERIALS AND METHODS

### 2.1 Ethics and informed consent

All participants provided written informed consent prior to participating in the investigation, and the study was approved by the *Comité d’éthique de la recherche de l’Institut universitaire de cardiologie et de pneumologie de Québec – Université Laval* (CER: 21180) according to the principles established in the Declaration of Helsinki (except for registration in a database).

### 2.2 Participants

Ten moderately trained women were enrolled in this study (age: 27 ± 6 years, height: 1.64 ± 0.06 m, body mass: 59.9 ± 6.2 kg, body mass index: 22 ± 2 kg/m^2^, maximal oxygen uptake ($\dot{V}$O_2max_): 48.6 ± 3.8 ml·kg·min^−1^, training volume: 465 ± 159 min/week). All the participants trained in a variety of endurance‐based sports including cycling (*n* = 1), triathlon (*n* = 4), mountain biking (*n* = 1), running (*n* = 3), and cross‐country skiing (*n* = 1). All participants were free from any medical conditions, demonstrated a normal 12‐lead electrocardiogram (ECG), and were not taking any medications. Women were either taking oral contraceptive continuously since >1 year (*n* = 2), wearing an intrauterine device (*n* = 2), or were tested during menses or the early follicular phase (day 1‒10) of their menstrual cycle (*n* = 6).

### 2.3 Experimental protocol

This study was part of a larger study examining the influence of an elevated cardiorespiratory fitness on dynamic cerebral autoregulation in young healthy women (Labrecque et al., 2019). However, the current question was determined a priori and was prospectively studied as a separate question. Anthropometrics measurements, $\dot{V}$O_2max_, as well as hemodynamics of the MCA have previously been published (Labrecque et al., 2019). Participants visited the laboratory on two occasions to perform: (a) an incremental cycling protocol for $\dot{V}$O_2max_ determination, and (b) anthropometrics, resting measurements, and systemic and cerebral hemodynamics measurements during a 30‐s high‐intensity exercise bout followed by a standardized recovery. Participants were asked to avoid exercise training for at least 12 hr, as well as alcohol, drugs and caffeine consumption for 24 hr before each visit. All sessions and evaluations were executed in the exact same order for all participants and there was at least 48 hr between testing sessions.

### 2.4 Measurements

#### 2.4.1 Systemic hemodynamics

Heart rate (HR) was measured using a 5‐lead ECG. Beat‐to‐beat BP was measured by the volume‐clamp method using a finger cuff (Nexfin, Edwards Lifesciences). The cuff was placed on the right middle finger and referenced to the level of the heart using a height correct unit for BP correction. MAP was obtained by integration of the pressure curve divided by the duration of the cardiac cycle. This method has been shown to reliably index the dynamic changes in beat‐to‐beat BP which correlate well with the intra‐arterial BP recordings and can be used to describe the dynamic relationship between BP and cerebral blood velocity (Omboni et al., 1993; Sammons et al., 2007).

#### 2.4.2 Blood velocity in middle and posterior cerebral arteries

MCAv_mean_ and PCAv_mean_ were monitored with a 2‐MHz pulsed transcranial Doppler ultrasound (Doppler Box; Compumedics DWL USA, Inc.). Identification and location of the left MCA and right PCA was determined using standardized procedures (Willie et al., 2011). Probes were attached to a headset and secured with a custom‐made headband and adhesive conductive ultrasonic gel (Tensive, Parker Laboratory) to ensure a stable position and angle of the probe throughout testing.

#### 2.4.3 End‐tidal partial pressure of carbon dioxide

End‐tidal partial pressure of carbon dioxide (P_ET_CO_2_) was continuously measured during all the tests through a breath‐by‐breath gas analyzer (Breezesuite, MedGraphics Corp.) calibrated to known gas concentrations following manufacturer instructions before each evaluation.

#### 2.4.4 Data acquisition

For each assessment, signals (except for P_ET_CO_2_) were analog‐to‐digital converted at 1 kHz via an analog‐to‐digital converter (Powerlab 16/30 ML880; ADInstruments, Colorado Springs) and stored for subsequent analysis using commercially available software (LabChart version 7.1; ADInstruments). P_ET_CO_2_ was time‐aligned with the other signals.

### 2.5 Visit 1

#### 2.5.1 Maximal oxygen consumption ($\dot{V}$O_2max_)

$\dot{V}$O_2max_ was determined during a progressive ramp exercise protocol performed on an electromagnetically braked upright cycle ergometer (Corival, Lode). Following 3min of rest, the evaluation started with 1min of unloaded pedaling followed by an incremental ramp protocol (from 22 to 25 W/min according to participant's history of training) to volitional exhaustion. Expired air was continuously recorded using a breath‐by‐breath gas analyzer (Breezesuite, MedGraphics Corp.) for determination of $\dot{V}$O_2_, carbon dioxide production ($\dot{V}$CO_2_), respiratory exchange ratio (RER: $\dot{V}$CO_2_/$\dot{V}$O_2_), and P_ET_CO_2_. Maximal $\dot{V}$O_2_ was defined as the highest 30‐s averaged $\dot{V}$O_2_, concurrent with a RER ≥ 1.15.

### 2.6 Visit 2

#### 2.6.1 Anthropometric measurements and resting hemodynamics

Height and body mass were measured in each participant. Resting hemodynamic measurements included MAP (volume‐clamp method using a finger cuff), which has been validated against intra‐arterial pressure (Omboni et al., 1993), heart rate (HR; ECG), MCAv_mean_, and PCAv_mean_, which were continuously monitored on a beat‐by‐beat basis during 5 min of seated rest. Cerebrovascular conductance index (CVCi; MCAv_mean_ or PCAv_mean_/MAP) and its reciprocal, resistance (CVRi; MAP/MCAv_mean_ or PCAv_mean_) were then calculated. P_ET_CO_2_ (gaz analyzer) was continuously monitored on a breath‐by‐breath basis. The average values of the last minute of recording represented the baseline.

#### 2.6.2 High‐intensity exercise bout

The high‐intensity exercise bout was performed on the same cycle ergometer used for assessment of $\dot{V}$O_2_max (Corival, Lode). Participants performed a 3‐min warm‐up at 50 W followed by 3min of passive rest during which they were asked to relax and avoid talking. Then, participants had to cycle for 30 s at the maximal workload achieved at $\dot{V}$O_2_max (240 ± 32 W) at a maximal pedaling rate of 110 rpm. They were asked to avoid Valsalva maneuver and to pay caution not to move their head and squeeze their fingers to optimize the quality of transcranial Doppler ultrasound and photoplethysmography signals. Maximal workload was reached within ~7 s for each participant (time required for the cycling ergometer to reach target workload). Upon exercise termination, participants were asked to completely stop pedaling, and to remain still on the ergometer for 3min of passive recovery.

Data were acquired continuously and averaged into 1‐s bins. Baseline data were averaged over the last 60 s of seated ergometer rest (after the warm‐up). During cycling exercise, peak MCAv_mean_ and PCAv_mean_ and their corresponding MAP, as well as the peak reduction in P_ET_CO_2_, were identified. The time delay before the onset of the regulatory response following onset of exercise, for example, when CVRi begins to continuously increase without any subsequent transient reduction in response to exercise‐induced increase in MAP, was also calculated. End‐exercise data were averaged over the last 5 s of exercise. During recovery, peak MCAv_mean_ and PCAv_mean_ during the initial 60 s following the end of exercise, their corresponding MAP, and the peak increase in P_ET_CO_2_ were identified.

### 2.7 Statistical analysis

The normal distribution of data was confirmed using Shapiro–Wilk normality tests. Differences between MCAv_mean_ and PCAv_mean_ were analyzed with paired *t* tests. Wilcoxon test was used if data were not distributed normally. Two‐way repeated measure ANOVAs (factors: artery and time as repeated measure) were performed to compare responses of each variable (MCAv_mean_, PCAv_mean_, MAP, and P_ET_CO_2_) at specific time points of interest during the 30‐s high‐intensity exercise bout [i.e., baseline, peak blood velocities (MCAv_mean_ and PCAv_mean_) during exercise, end‐exercise, peak blood velocities (MCAv_mean_ and PCAv_mean_) during recovery]. Statistical significance was established a priori at *p* < .05 for all two‐tailed tests. Data are expressed as mean ± standard deviation.

## 3 RESULTS

One participant was excluded from the high‐intensity exercise bout analysis, because of inconsistency in the MAP recording, and we were unable to measure PCAv_mean_ in two participants. The final sample size for the high‐intensity exercise bout analysis was *n* = 10 for MCAv_mean_, *n* = 8 for PCAv_mean_, *n* = 9 for MAP and CVRi/CVCi in the MCA, and *n* = 7 for CVRi/CVCi in the PCA.

### 3.1 Participants baseline characteristics and resting values

Baseline characteristics and systemic and cerebral hemodynamics are reported in Table 1. MCAv_mean_ was higher compared to PCAv_mean_ (72 ± 7 vs. 41 ± 5 cm·s^‐1^; *p* < .0001). CVRi was lower (1.46 ± 0.17 vs. 2.71 ± 0.41 mmHg·cm·s^−1^; *p* = .0001), whereas CVCi was higher in the MCA (0.69 ± 0.07 vs. 0.38 ± 0.07 cm·s^−1^·mmHg^−1^; *p* = .02).

**TABLE 1:** Baseline characteristics and resting seated values

Baseline characteristics	
Age (years)	27 ± 6
Weight (kg)	59.9 ± 6.2
Height (m)	1.64 ± 0.06
Body mass index (kg/m^2^)	22 ± 2
Maximal O_2_ consumption (mL/kg·min^−1^)	48.6 ± 3.8
Resting values	
*n*	11
Heart rate (bpm)	75 ± 16
Mean arterial pressure (mmHg)	105 ± 11
Cardiac output (L/min)	5.3 ± 1.3 (*n* = 9)
Middle cerebral artery mean blood velocity (cm·s^−1^)	72 ± 7
Posterior cerebral artery mean blood velocity (cm·s^−1^)	41 ± 5 (*n* = 8)
MCA cerebrovascular resistance index (mmHg·cm·s^−1^)	1.46 ± 0.17 (*n* = 9)
PCA cerebrovascular resistance index (mmHg·cm·s^−1^)	2.71 ± 0.41 (*n* = 7)
MCA cerebrovascular conductance index (cm·s^−1^·mmHg^−1^)	0.69 ± 0.07 (*n* = 9)
PCA cerebrovascular conductance index (cm·s^−1^·mmHg^−1^)	0.38 ± 0.07 (*n* = 7)
End‐tidal carbon dioxide partial pressure (mmHg)	37 ± 2

Note

Data are presented as mean ± *SD*.

### 3.2 Acute systemic and cerebrovascular responses during and following 30 s of high‐intensity exercise

**FIGURE 1:**
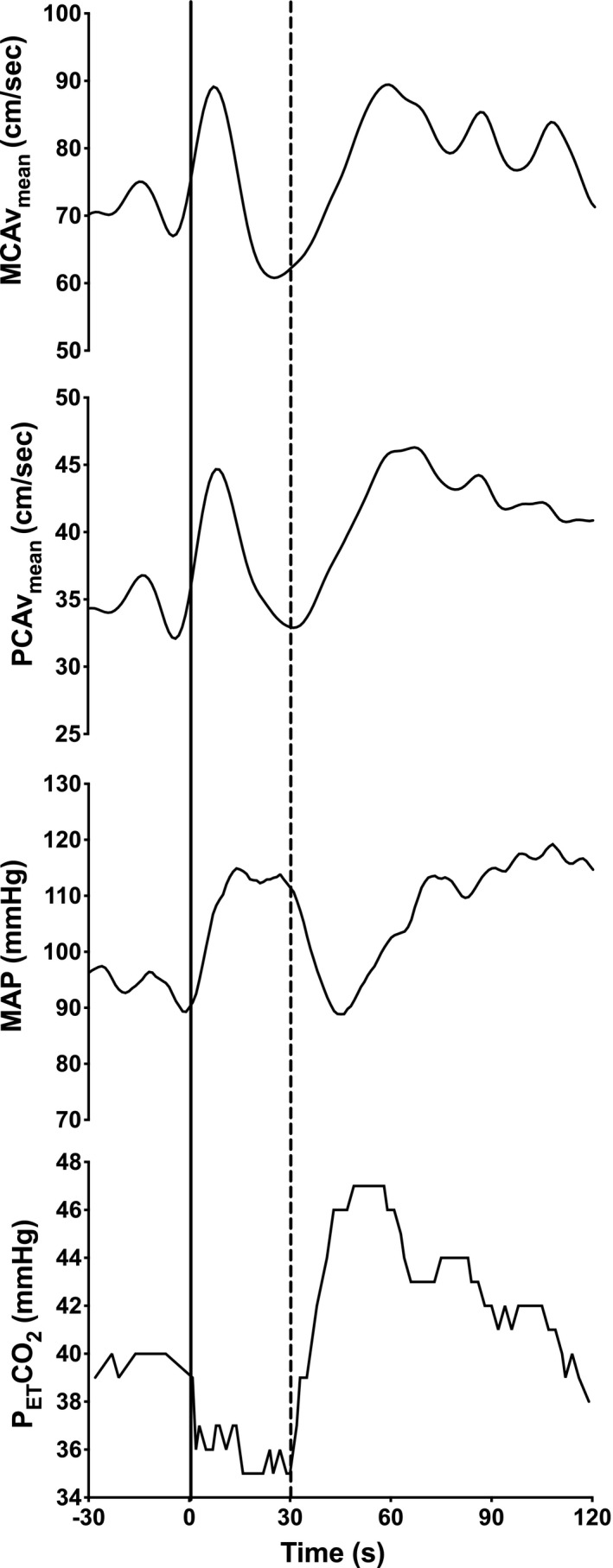
Averaged continuous responses of mean arterial pressure (MAP), blood velocity in the middle (MCA), and posterior (PCA) cerebral arteries and end‐tidal carbon dioxide (P_ET_CO_2_) during 30‐s seated rest, 30‐s high‐intensity exercise, and the following recovery. Time 0 (full line) indicates the beginning of the high‐intensity exercise bout and time 30 (dashed line) indicates the beginning of the recovery period

Temporal responses of MAP, MCAv_mean_, PCAv_mean_, and P_ET_CO_2_ during and following the high‐intensity exercise bout from one representative participant are depicted in Figure 1. From baseline to peak value during the 30‐s exercise bout, MCAv_mean_ and PCAv_mean_ increased by 19 ± 10% (10 ± 8 s after the onset of exercise) and 21 ± 14% (9 ± 6 s after the onset of exercise), respectively (ANOVA time effect *p* < .0001; Table 2). The time delay before the onset of the regulatory response following the start of exercise was not different between the MCA and PCA (11 ± 4 vs. 11 ± 3 s; *p* > .99). Then, MCAv_mean_ and PCAv_mean_ returned toward baseline values at the end of the high‐intensity exercise bout.

**TABLE 2:** Normalized responses of blood velocity in the middle and posterior cerebral arteries and mean arterial pressure at each stage of the high‐intensity exercise bout

	High‐intensity exercise bout stages				*p* values		
	Baseline	Peak	End	Recovery	Time	Artery	Interaction
Blood velocities (%)							
MCA	100 ± 0	119.4 ± 10.0	101.9 ± 12.6	143.2 ± 15.4	**<0.0001**	0.7205	0.7714
PCA	100 ± 0	120.8 ± 14.3	107.4 ± 14.5	142.0 ± 15.2			
MAP (mmHg)							
MCA*	105.4 ± 10.7	113.9 ± 13.1	122.6 ± 12.7	119.6 ± 13.4	**<0.0001**	0.9460	0.5190
PCA*	105.6 ± 12.1	110.4 ± 18.1	123.5 ± 13.7	123.6 ± 14.0			

Note

Data are presented as mean ± *SD*.

Abbreviations: MAP, Mean arterial pressure; MCA, middle cerebral artery; PCA, posterior cerebral artery.

* Indicates values of MAP at each exercise stage for both MCA and PCA.

Bold values were used to denote statistical significance.

During recovery and compared to baseline values, MCAv_mean_ and PCAv_mean_ progressively increased by 43 ± 15% (44 ± 9 s following the end of exercise) and by 42 ± 15% (48 ± 7 s following the end of exercise), respectively (ANOVA time effect *p* < .0001; Table 2). CVCi started to increase 4 ± 2 s following the onset of recovery for both MCA and PCA (*p* = .59) and increased by 61 ± 44% for MCA and by 40 ± 20% for PCA (*p* = .30) compared to the values averaged over the last 5 s of the high‐intensity exercise. These peak values were reached 27 ± 13 s (MCA) and 31 ± 8 s (PCA; *p* = .26) into recovery.

From baseline to peak MCAv_mean_, MAP increased by 9 ± 2 mmHg (from 105 ± 11 to 114 ± 13 mmHg), while MAP increased by 4 ± 6 mmHg (from 106 ± 12 to 110 ± 18) from baseline to peak PCAv_mean_ (Table 2). Then, MAP continued to increase until the end of exercise (ANOVA time effect *p* < .0001; Table 2). During recovery, MAP remained higher than baseline (ANOVA time effect *p* < .0001) notwithstanding an acute reduction after the end of the high‐intensity exercise (−22 ± 6 mmHg in 16 ± 6 s).

Compared to baseline, P_ET_CO_2_ had a maximal decrease (−3 ± 2 mmHg; *p* < .0001) 9 ± 5 s following the onset of exercise, but returned to baseline values at the end of the 30‐s exercise bout (Table 2). During recovery, P_ET_CO_2_ significantly increased to reach its peak value (+11 ± 3 mmHg) 23 ± 12 s following the end of exercise. At the end of the 3‐min recovery, the P_ET_CO_2_ averaged over the last 10 s was still 9% higher than pre‐exercise baseline values.

## 4 DISCUSSION

This study provides a detailed temporal description of cerebral blood velocity responses in the MCA and PCA to a 30‐s high‐intensity exercise and its time‐course recovery in young fit women. The main findings of this study are that: (a) the acute cerebral blood velocity responses to a high‐intensity exercise bout are biphasic, characterized by a rapid increase relative to baseline followed by a return toward baseline values at the end of exercise in both the MCA and PCA; and (b) the passive recovery period following a 30‐s high‐intensity exercise is characterized by similarly large elevations in MCAv_mean_ and PCAv_mean_ relative to the end of exercise.

### 4.1 Biphasic responses of MCAv_mean_ and PCAv_mean_ during one high‐intensity exercise bout

During progressive cycling exercise to exhaustion, cerebral blood flow increases up to ~70% of $\dot{V}$O_2_max (Madsen et al., 1993; Smith et al., 2012). From this intensity threshold to maximal exercise, cerebral blood flow returns toward baseline values because of hyperventilation‐induced hypocapnia and consequent cerebral vasoconstriction. HIIT is distinctive from progressive aerobic exercise to exhaustion, as it includes short exercise bouts of near‐ to supra‐maximal intensity, associated with marked rapid increases in BP. Therefore, maximal BP values are reached sooner, and in a much more sudden manner during HIIT than progressive aerobic exercise to exhaustion (e.g., during a $\dot{V}$O_2_max protocol) (Calbet et al., 2015; Curtelin et al., 2018; Tsukamoto et al., 2018). Repetitive sudden elevations in MAP induced by acute high‐intensity exercise could be transmitted to the brain and could cause damage if not adequately buffered by neuroprotective mechanisms [reviewed in (Lucas et al. (2015)]. During a 30‐s Wingate test, the MAP of healthy men increases continuously during the exercise bout, whereas MCAv_mean_ displays a biphasic response, increasing abruptly in the beginning of the sprint, before returning to baseline values (Curtelin et al., 2018). Our results extend these descriptive findings for MCAv_mean_ and PCAv_mean_ in healthy fit women. Accordingly, there is a time period immediately after the onset of the high‐intensity exercise bout, during which the brain will be exposed to pressure‐passive elevations in CBV. In the current study, peak increases in MCAv_mean_ and PCAv_mean_ occurred ~10 s following exercise onset, before returning to baseline values at the end of the exercise bout. Interestingly, the time delay before the onset of the regulatory response following the beginning of exercise, that is the elevation in CVRi in the MCA and PCA, was 11 s in the current study. Even though it is recognized that dynamic cerebral autoregulation takes place in approximately 5 s during an abrupt hypotensive stimulus (Aaslid, Lindegaard, Sorteberg, & Nornes, 1989), no study, to our knowledge, examined dynamic cerebral autoregulation onset during a rapid and important rise in MAP and CBV caused by high‐intensity exercise. Dynamic cerebral autoregulation evaluated during hypertensive stimuli has been characterized during progressive MAP increases using handgrip (Caldas et al., 2017), drug infusions (Ogoh et al., 2011), or cold pressor test (Vianna, Sales, & Nobrega, 2012). Dynamic cerebral autoregulation onset (i.e., vasoconstrictor response) following high‐intensity exercise and the associated quick elevation in MAP is currently absent from the existing literature. Further studies will be necessary to better understand whether the time delay before the onset of cerebral autoregulation has an impact on the amplitude of CBV change at the beginning of high‐intensity exercise. In addition, there is also a delay before any change in CO_2_ influences the cerebrovasculature [reviewed in Hoiland, Fisher, & Ainslie (2019)]. Accordingly, the lowering in P_ET_CO_2_ (−3 ± 2 mmHg compared to baseline in 9 ± 5 s) observed early into exercise most likely contributed to the reduction in cerebral blood velocity later during the exercise bout.

Taken together, these findings suggest that during a 30‐s high‐intensity exercise bout performed at maximal workload, the cerebrovasculature of healthy young fit women is challenged by an important increase in MAP soon after the onset of exercise, which could be the consequence of a delay in the onset of the regulatory response. Then, following an increase in cerebrovascular resistance and hypocapnia‐induced cerebral vasoconstriction, cerebral blood velocity returns toward baseline value at the end of the exercise bout. Further research on the “time window” during which CBV seems to be more pressure‐passive at the beginning of intense exercise is needed to better understand if it increases the risk of brain hyperperfusion, especially if this time window is extended. We speculate that this time period where CBV passively increases with rapid surges in BP at the onset of high‐intensity exercise in healthy fit women will be longer in patients with delayed onset of regulatory response or attenuated cerebrovascular reactivity to CO_2_. Considering the popularity of HIIT in various clinical populations with attenuated cerebral autoregulation and cerebrovascular reactivity to CO_2_ (Last et al., 2007; SS Meel‐van den Abeelen, Lagro, & Beek, 2014), more research examining the cerebrovascular responses during various high‐intensity exercise prescriptions in these clinical populations is thus warranted.

### 4.2 Important elevations in MCAv_mean_ and PCAv_mean_ following one high‐intensity exercise bout

The cerebrovascular function seems to be preserved after the cessation of moderate‐intensity exercise (Steventon et al., 2018; Willie, Ainslie, Taylor, Eves, & Tzeng, 2013). To the best of the authors’ knowledge, no previous studies evaluated CBV during the immediate and extended recovery period following a 30‐s high‐intensity exercise in healthy young women. In the current study, the transition from high‐intensity exercise to the onset of passive recovery led to immediate hemodynamic changes. Despite a transient reduction in MAP (~20 mmHg in 15 s), MCAv_mean_ (+43%), and PCAv_mean_ (+42%) considerably increased, which was most likely driven by an elevation in P_ET_CO_2_. Increases in CVCi (MCA: +61 ± 44%; PCA: 40 ± 20%) support this assumption. There is no evidence that an acute elevation in cerebral blood velocity associated with hypercapnia during a passive recovery period is harmful for healthy brain vessels. Yet, such acute and rapid elevations in CBV could increase the risk of hyperperfusion injury in individuals with impaired cerebrovascular function or diseased brain vessels. Further research will be necessary to evaluate whether repetitive and rapid cerebral blood flow elevations following high‐intensity exercise lead to beneficial adaptations or damaging consequences in patients with diseased cerebral vessels.

### 4.3 Perspectives

This study was designed to describe the cerebrovascular responses to one bout of high‐intensity exercise and the associated sudden elevation in MAP. However, we acknowledge that this exercise stimulus does not represent a realistic HIIT session, during which multiple intervals are usually performed, interspersed with passive or active recovery. For example, in healthy children, repetitions of 1‐min intervals at 90% of maximal workload interspersed with 1‐min active rest periods resulted in a ~11% decrease in MCAv_mean_ from the first to the last high‐intensity exercise interval (Tallon, Simair, Koziol, Ainslie, & McManus, 2019). Tsukamoto et al. (2018) also evaluated changes in MCAv_mean_ during a HIIT session, which included 4 x 4 min of aerobic exercise performed at 80‒90% maximal power output interspersed with 3‐min rest periods at 50‒60% maximal workload. MCAv_mean_ from the 4 intervals (averaged over each interval duration) did not change from baseline in these young healthy men. Reduction in P_ET_CO_2_ with the addition of exercise intervals and recovery periods could partly explain why HIIT did not increase CBV notwithstanding elevations in MAP in this study (Tsukamoto et al., 2018). Further research is needed to better understand the respective roles of CO_2_ and other important determinants, such as dynamic cerebral autoregulation, in the cerebral blood flow response to different types and durations of acute HIIT sessions and related (passive or active) recovery, in order to optimize cerebrovascular adaptation to HIIT.

### 4.4 Limitations

Some limitations to our study need to be acknowledged and further discussed. Only young healthy fit women participated to this study and the results cannot be generalized to other populations (such as men, older individuals, or hypertensive patients). Moreover, the cerebrovascular responses observed for a 30‐s high‐intensity exercise bout is not generalizable to other intensities or durations of exercise. The findings cannot be assumed to be similar during a complete HIIT session where multiple exercise bouts are repeated. Furthermore, since we used a passive recovery, our results can neither be applicable to active recovery.

BP was measured noninvasively by finger photoplethysmography. While specific instructions were given to participants in order to avoid squeezing their fingers to optimize the signal, we acknowledge that BP may have been different compared to an invasive BP monitoring. Further to this point, MCAv_mean_ and PCAv_mean_ were monitored with transcranial Doppler ultrasound, and would be representative of flow only if the diameter of the arteries remains stable. The mean reduction in P_ET_CO_2_ during the 30 s of intense exercise was –3 ± 2 mmHg; (range 0‒6 mmHg) compared to baseline. Based on previous work (Ainslie & Hoiland, 2014), intracranial vessels’ diameter probably changed minimally. As for absolute changes in P_ET_CO_2_ during recovery, the maximal elevation in P_ET_CO_2_ was +11 ± 3 mmHg; (range 7–16 mmHg) above baseline values. This variation in P_ET_CO_2_ most likely influenced the diameter of intracranial vessels. Accordingly, CBV is certainly underestimating cerebral blood flow (Ainslie & Hoiland, 2014). Taken together, we assume a major part of the CBV increase reported during intense exercise is explained by a rapid surge in MAP. However, P_ET_CO_2_ is a major confounding factor during recovery.

Since female participants in this study were either taking oral contraceptives continuously (*n* = 2), having an intrauterine device (*n* = 2), or evaluated during days 1‒10 of their menstrual cycle (*n* = 6), we are unable to ascertain if MCAv_mean_ and PCAv_mean_ responses to high‐intensity exercise were influenced by the oscillatory nature of hormones throughout the menstrual cycle. Further research is warranted to determine the specific effects the stages of the menstrual cycle play on these measures.

## 5 CONCLUSION

These results suggest that in healthy young fit women, both MCAv_mean_ and PCAv_mean_ quickly increase at the onset of a 30 s of high‐intensity exercise performed at maximal workload, before returning to baseline values through the end of the exercise. During recovery, cerebral blood velocity augments in both arteries, along with P_ET_CO_2_.

## CONFLICT OF INTEREST

No conflicts of interest, financial, or otherwise, are declared by the author(s).

## AUTHORS CONTRIBUTION

P.B. contributed to the original idea of the study; L.L., K.R, and S.I, contributed to data collection; L.L. contributed to data analyses; L.L, F.B., and P.B. contributed to data interpretation; L.L., A.D., and P.B drafted the article. All authors provided approval of the final article.
